# Opportunities, challenges, and policy implications of the aerobiome paradigm shift

**DOI:** 10.1128/msphere.00203-25

**Published:** 2025-08-07

**Authors:** Martin F. Breed, Craig Liddicoat, Xin Sun, Sunita Ramesh, Scott Hawken, Kevin Lee, Joel Brame, Nicole W. Fickling, Emma Kuhn, Claire Hayward, Sonali Deshmukh, Kate Robinson, Christian Cando-Dumancela, Jake M. Robinson

**Affiliations:** 1 College of Science and Engineering, Flinders University 117627, Bedford Park, South Australia, Australia; 2 The Aerobiome Innovation and Research Hub, Flinders University 1065, Bedford Park, South Australia, Australia; 3 Key Laboratory of Urban Environment and Health, Ningbo Observation and Research Station, Institute of Urban Environment, Chinese Academy of Sciences 85406 , Xiamen, China; 4 Zhejiang Key Laboratory of Urban Environmental Processes and Pollution Control, CAS Haixi Industrial Technology Innovation Center in Beilun, Ningbo, China; 5 University of Chinese Academy of Sciences 74519 , Beijing, China; 6 School of Architecture and Civil Engineering, The University of Adelaide 443979 , Adelaide, South Australia, Australia; 7 Department of Food Science and Microbiology, School of Science, Auckland University of Technology 682623 , East Auckland City, New Zealand; 8 School of Biotechnology and Biomolecular Sciences, University of New South Wales 98492, Kensington, New South Wales, Australia; 9 Environmental Health, College of Science and Engineering, Flinders University 117627, Adelaide, South Australia, Australia; Virginia-Maryland College of Veterinary Medicine, Blacksburg, Virginia, USA

**Keywords:** aerobiome, urban microbiome, bioaerosols, microbiome, urban ecology, AIR Hub

## Abstract

Historically, bioaerosol research has focused on identifying and mitigating the harmful effects of airborne pathogens and particles. These bioaerosols—including bacteria, viruses, fungal spores, and non-biological particles, such as particulate matter up to 2.5 µm (PM2.5)—pose substantial risks to human and ecosystem health. They can contribute to diseases and adverse outcomes in humans, animals, plants, and their associated microbial communities. Researchers have concentrated on understanding transmission mechanisms, detecting and quantifying these agents, and developing control strategies. However, a recent paradigm shift in aerobiome thinking highlights the importance of beneficial bioaerosols in maintaining ecosystem and human health. Beneficial bioaerosols, such as salutogenic (health-promoting) microbiota, phytoncides (plant-derived organic compounds), pheromones, and potentially “aeronutrients” contribute to human health modulation and important ecosystem processes. This dual nature of bioaerosols necessitates a holistic approach to promote beneficial components while mitigating harmful ones. Here, we introduce a recently established initiative called the Aerobiome Innovation and Research Hub (AIR Hub), which aims to advance this interdisciplinary research. We call for action to further understand and leverage the beneficial biological components of air for both human and ecosystem health and present the results of an AIR Hub workshop “reverse brainstorming” session to identify novel opportunities and challenges. These include key barriers to advancing aerobiome science, such as poor communication, methodological complexity, and fragmented regulation. Solutions focused on clearer definitions, improved research methods, targeted communication, and stronger policy engagement. Finally, we present the key policy implications of advancing this aerobiome paradigm shift.

## INTRODUCTION

Historically, bioaerosol research has predominantly focused on identifying and mitigating the harmful effects of airborne pathogens and particulate matter (1, 2). This emphasis is well reflected in reviews, such as Nazaroff (3), which provide a comprehensive overview of airborne microbial exposure risks in indoor environments, particularly in relation to human health protection. Harmful bioaerosols, which include bacteria, viruses, fungal spores, and pollen, can pose significant risks to human health, ecosystem processes, and agricultural productivity (4, 5). For instance, airborne pathogens can lead to respiratory diseases, allergies, and infections in humans, while also causing widespread damage to wild and agricultural plants and wild and domestic animals (6, 7). For decades, researchers have concentrated on understanding the transmission mechanisms of these harmful agents, developing methods to detect and quantify their presence, and creating strategies to control their spread (8). This focus was driven by the urgent need to protect public health and ensure food security. Major outbreaks of diseases, such as tuberculosis, influenza, coronavirus disease 2019 (COVID-19), and agricultural blights have underscored the importance of controlling harmful bioaerosols (9).

Research in this area is vital. However, there has been a recent paradigm shift in thinking about bioaerosols (10). Researchers now recognize the importance of beneficial bioaerosols in maintaining ecosystem and human health (11, 12). This new perspective emphasizes the need to understand and harness the beneficial aspects of bioaerosols, such as their role in nutrient cycling, plant growth, and human immune system modulation. Beneficial bioaerosols, including non-pathogenic bacteria, fungal spores, phytoncides (plant-derived organic compounds), and pheromones, can contribute to environmental and human health in various ways. For instance, phytoncides (e.g., alpha and beta-pinene) are known to reduce blood pressure, enhance NK cell activity in people (13, 14), and improve sleep in mouse models (15). The concept of “aeronutrients” (airborne nutrients or bioactive molecules, such as manganese and iodine that may have salutogenic effects) was also recently discussed (16). It is also known that floral (i.e., organic) cues emitted by plants are important in the pollination process, and pollution can adversely impact this relationship (17). Moreover, bumble bees’ exposure to different environmental conditions and microbiota influences their gut-community composition, with potential implications for their health (18).

Additionally, exposure to diverse microbial communities in the air can help train and regulate the human immune system, potentially reducing the prevalence of allergies and autoimmune diseases (19). Exposure to urban house dust drives an allergic Th1-type immune response, while exposure to biodiverse rural dust drives a Th2-type anti-inflammatory immune response (11). Exposure to microbiota from natural environments (via touch and undefined exposure pathways) is known to increase species richness in the human microbiome and enhance immunoregulation (20). Moreover, being close to a microbially diverse indoor green wall is associated with an increase in beneficial skin microbiota (21).

Understanding the dual harmful and beneficial nature of bioaerosols is essential for developing comprehensive strategies to improve public and ecosystem health. The correlations between these groups of bioaerosols must be understood to avoid inadvertently promoting harmful bioaerosols in an attempt to safeguard beneficial bioaerosols. By promoting beneficial bioaerosols and mitigating harmful ones, we can create healthier environments for humans and ecosystems alike. Recent reviews have highlighted the critical role of the microbiome in linking human, animal, and environmental health, reinforcing the value of a One Health approach to microbiome research (22). This integrative framing supports our call for interdisciplinary collaborations and systems-level thinking in aerobiome science.

To operationalize this holistic thinking, interdisciplinary collaborations are required. To this end, we founded the Aerobiome Innovation and Research Hub (AIR Hub; https://www.aerobiome.org). The AIR Hub is currently made up of 16 academics and practitioners from various disciplines (e.g., microbial ecology, restoration ecology, immunology, landscape architecture, soil biology, bioinformatics, environmental health, and molecular biology) that influence bioaerosol research and this holistic way of thinking about air and its biological components (Table 1).

**TABLE 1:** Glossary of terms

Term	Definition
Aerobiome	The collection of microorganisms (and their theater of activity) in a given air space.
Bioaerosols	Airborne particles that contain or are derived from living organisms, such as bacteria, viruses, pollen, and fungal spores.
Biodiversity	The variety and variability of life forms within a given ecosystem, region, or the entire planet, encompassing diversity in species, genes, traits, functions, and ecosystems.
Biogenic	Originating from biological processes, referring to substances or particles produced by living organisms.
Environmental health	The branch of public health that focuses on the interactions between people and their environment, aiming to prevent health problems related to environmental exposures.
Landscape architecture	The art and science of designing, developing, and managing outdoor spaces, environments, and public spaces to achieve ecological, social, and aesthetic outcomes.
Particulate matter	A mixture of solid particles and liquid droplets found in the air, which can include dust, dirt, soot, and smoke, and can have adverse health effects when inhaled.
Restoration ecology	The science of restoring degraded, damaged, or destroyed ecosystems.
Volatile organic compounds	Organic chemicals that have a high vapor pressure at room temperature, contributing to air quality and potentially impacting health and the environment.

While a unified theoretical framework for beneficial bioaerosol function is still emerging, current evidence points to several plausible and interacting biological mechanisms. These include modulation of immune function via exposure to environmental microbial diversity, enhancement of microbiome richness through airborne microbial transfer, physiological responses to plant-derived volatiles and aeronutrients, and broader ecosystem-level processes, such as pollination and soil–plant–air microbial feedbacks (14, 17, 19, 20). Together, these mechanisms suggest a need for an integrative model that captures the multi-scalar and cross-kingdom nature of bioaerosol impacts. We anticipate that ongoing interdisciplinary collaboration will help formalize a conceptual structure to support both scientific investigation and policy translation in this area.

Here, we introduce the Aerobiome Innovation and Research Hub (AIR Hub), which aims to advance this interdisciplinary research. We call for action to further understand and leverage the beneficial biological components of air for both human and ecosystem health and present the results of a “reverse brainstorming” session to identify opportunities and challenges. Finally, we present the key policy implications of advancing this aerobiome paradigm shift.

## THE MISSION

**Fig 1:**
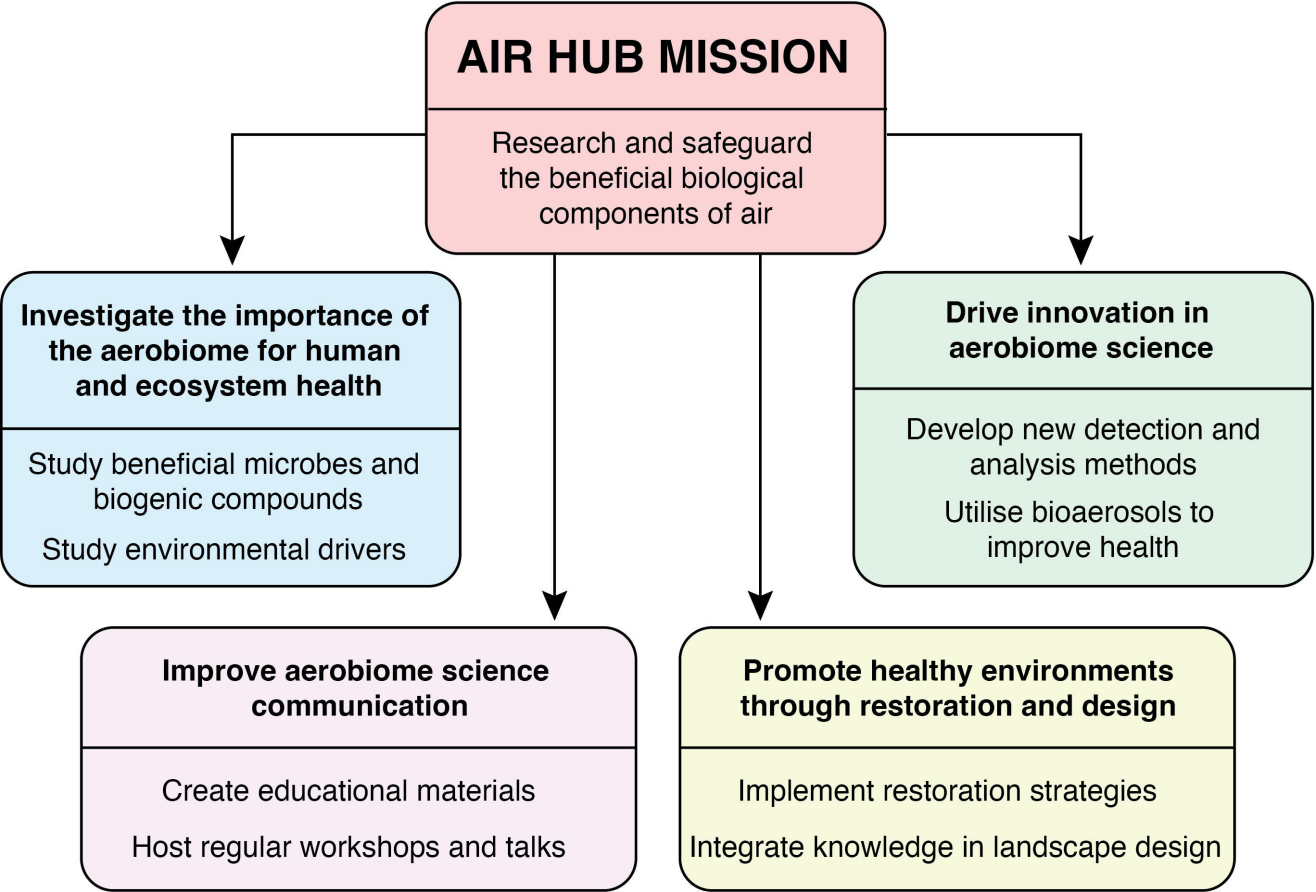
The AIR Hub mission components.

The core aim is to research and safeguard the beneficial biological components of air and encompasses four key objectives (Fig. 1).

### Investigate the importance of the aerobiome for human and ecosystem health

Researching the critical role that airborne microbial communities play in maintaining human health and ecosystem stability. This includes studying how beneficial microbes and biogenic compounds in the air can enhance immune system function, promote mental well-being, and support biodiversity. We also aim to understand how biodiversity and environmental drivers shape the aerobiome.

### Drive innovation in aerobiome science

Fostering cutting-edge research and technological advancements in aerobiome science. By developing new methods for detecting, analyzing, and utilizing beneficial bioaerosols, the aim is to advance the understanding and application of these invisible components in improving human and ecosystem health.

### Improve aerobiome science communication

Effective communication of scientific findings is essential. We should strive to enhance public and scientific community awareness of the importance of the aerobiome. This involves creating accessible educational materials, engaging with the media and hosting workshops/talks to share the latest research insights and innovations.

### Promote healthy environments through restoration and design

We advocate for and implement strategies to enhance environmental health through ecosystem restoration and biodiversity-positive landscape design. By integrating knowledge of beneficial bioaerosols into environmental management practices, we can create healthier, more resilient ecosystems and human communities.

## WHAT IS HEALTHY AIR?

**Fig 2:**
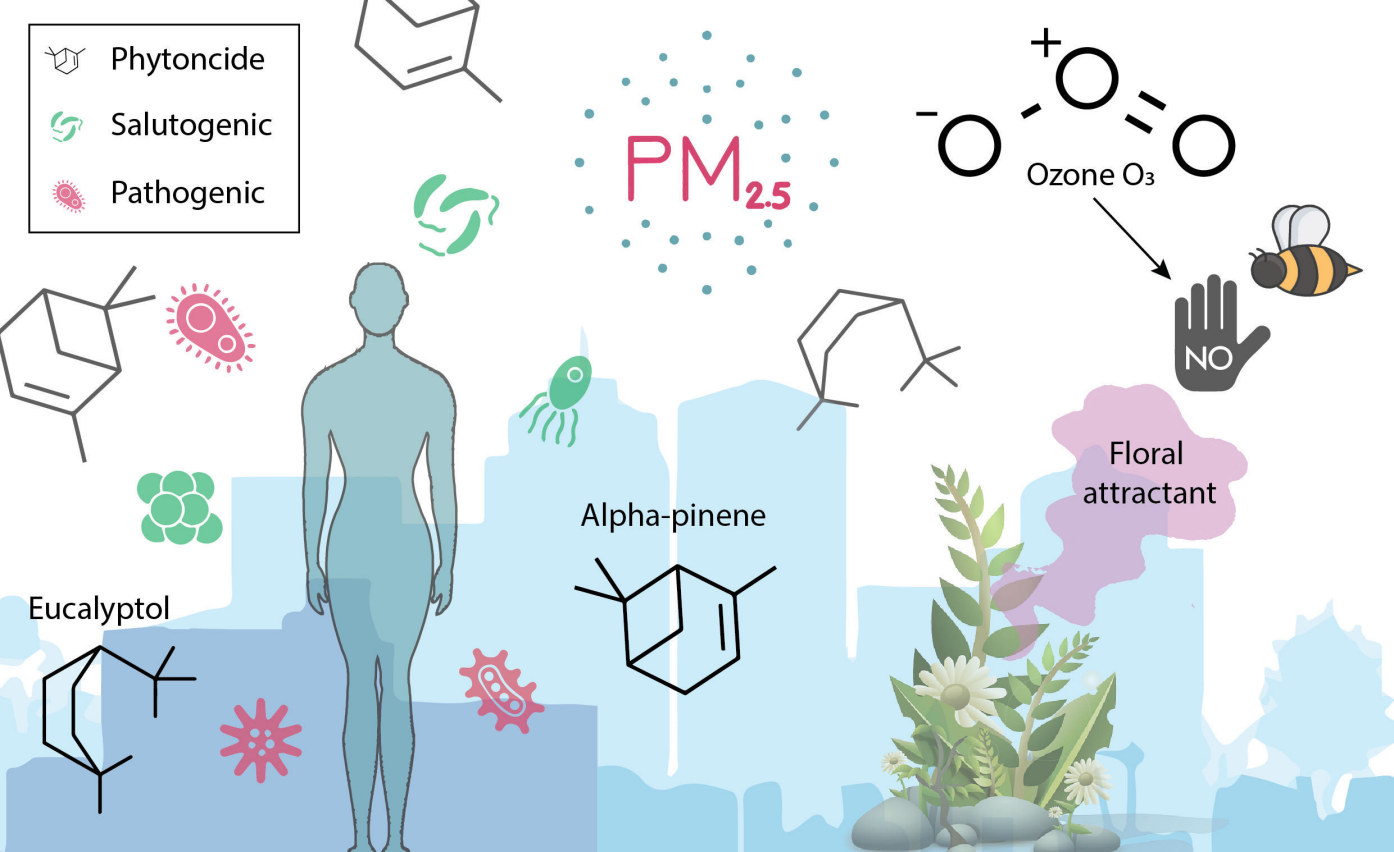
A shift in thinking about bioaerosols—taking a holistic view of the biological components of air (both negative and positive) and how they interact with human and ecosystem health in pathogenic (health-demoting) and salutogenic (health-promoting) ways. This includes considerations for the non-biological particles (e.g., PM) that interact with the bioaerosols.

Defining “healthy air” can be challenging. One might typically define it as air that is free from pollutants and harmful substances that can negatively impact human health and ecosystems. However, drawing from modern public health narratives, “health” is considerably more than “the absence of disease” (23). Healthy air might consist of the appropriate balance of gases, including oxygen, nitrogen, and trace amounts of other gases, without excessive levels of pollutants, such as particulate matter (PM), nitrogen oxides (NOx), sulfur dioxide (SO_2_), carbon monoxide (CO), volatile organic compounds (VOCs), and ozone (O_3_). Given the recent paradigm shift in thinking about many microorganisms and biogenic compounds as having health-promoting properties, a definition of healthy air might then also include some consideration for the presence, abundance, and diversity of these constituents (Fig. 2). Further challenges involve the types and concentrations of pollutants, which vary widely depending on location, weather, and human activities (24). Urban areas may have higher levels of vehicular and industrial emissions, while rural areas might be more affected by agricultural activities and natural sources like dust or wildfires (25).

Different pollutants also have different health impact thresholds. For example, particulate matter (PM_2.5_) is harmful at lower concentrations compared to larger particles (PM_10_) (26). Determining safe exposure levels for all pollutants simultaneously is complex because they interact and can have cumulative or synergistic effects (27). Moreover, the health impacts of air pollution vary among individuals based on age, health status, genetic factors, and lifestyle (28). Vulnerable populations, such as children, the elderly, and those with pre-existing health conditions, are more susceptible to the adverse effects of poor air quality (29). Some species might tolerate or even prefer certain chemical parameters considered toxic to others. Furthermore, air quality standards that are appropriate for one region may not be suitable for another due to differences in local climate, geography, industrial activity, and baseline pollution levels. Thus, defining healthy air requires a contextual approach that considers these regional variations.

A workshop on defining “healthy air” coordinated by BioAirNet, a UK Research & Innovation (UKRI) Strategic Priorities Fund (SPF) clean air program (https://bioairnet.co.uk/), resulted in a white paper being produced, with the following conclusion:

“The idea of ‘clean’ air does not fit with the ever-present nature of bioaerosols. Current research supports the concept that both outdoor and indoor air contains many different types of bioaerosols that can both benefit and harm our health. Based on this existing knowledge, we propose that healthy air comprises a wide diversity of bioaerosols, while unhealthy air contains fewer types of bioaerosols in usually high concentrations” (30).

Given the emerging evidence for the importance of diverse exposures to certain environmental microorganisms and biogenic compounds (10), there is a need for interdisciplinary collaborations to gain further insights.

## THE AEROBIOME PARADIGM SHIFT “REVERSE BRAINSTORMING”

The inaugural AIR Hub workshop took place in December 2023. The workshop comprised a series of talks from academics and practitioners whose work relates to the aerobiome and air quality. This was followed by an evaluation of aerobiome sampling methods (Table 2) and a reverse brainstorming session to identify solutions to current and emerging issues that may hinder aerobiome research and innovation.

**TABLE 2:** Results from the aerobiome sampling evaluation^*a*^^,*b*^

Methodology	What it measures	Advantages	Disadvantages
Air impingement	All bioaerosols.	Culture independent. Program to sampling cycle.	Bubbles, osmotic stress, and foaming may impact cell viability. Liquid can evaporate over long sampling times. 10–12 L/min (low).
Air impaction	All bioaerosols.	An Anderson sampler can separate different bioaerosols based on size to replicate a human lung. Bioaerosol deposition onto agar or solid surface—diverse measurement. Affordable depending on impactor. Portable. Minimal handling after sampling.	Can be cost-prohibitive, depending on sampler choice Limited by culture medium portability. Agar plates can dry out over long sampling times. Bounce off bioaerosols. Colony overgrowth. Impaction can impact cell viability. High cell counts environments cause overgrowth.
Bertin Technologies Coriolis Micro sampler	All culturable bioaerosols. Wet sampling.	High volume (300 L/min). Particles captured in fluid prevent cell and genetic material degradation and ready for analysis.	Cost—unit is expensive. NiMH battery limited (1 h). Wet sampling and construction sensitive to challenging field environments.
Passive sampling	Bacteria, fungi, archaea.	Easy to use and can capture vertical stratification.	Time—need to leave for an extensive period. Contamination. Accuracy—at representing dynamic environments.
Bertin Technologies Coriolis Compact sampler	All culturable bioaerosols. Dry sampling.	Compact LiPo longer battery life (8 h at 20°C). High volume (50 L/min).	Cost—expensive. Dry air sampling—kill microbes/degrade genetic material.
Research International SASS 3100	All culturable bioaerosols. Dry sampling with single-use electret filters	Very rugged, easily field operable. Long battery life (25 h with rechargeable Li-ion battery). High volume (300 L/min). Programmable. Wireless control possible. ISO 14698-1 compliant.	Cost—very expensive (per unit and continual disposable filter cost). Dry sampling—kill microbes/degrade genetic material.
Filtration	All bioaerosols	Low cost. Diverse measurement method: Direct visualization via microscopy Liquid extraction for culture/molecular methods Diverse filter choice allowing for selective binding. Suitable for high cell count environments.	Qualitative studies. Cell viability loss due to drying out on the filter.
TRIO.BASAIRBIO TRIO		Portable three aspirating heads air sampler. 100 or 200 L/min flow rate model. Bluetooth capability for data transfer.	The aspirating chamber is suitable for 55 mm contact plates or 90 mm petri dishes (culture dependent).
BioCapt Single-Use Microbial Impactor/MiniCapt Mobile Microbial Air Sample		Adjustable height and diameter mechanism for versatile petri dish compatibility (86–92 mm). HEPA-filtered exhaust ensures no environmental contamination of critical control points. Autoclavable 316 L stainless steel impactor head. Sampling flow rate (25–100 L/min).	Culture dependent. Battery time (6 h [100 L/min]−12 h [25 L/min]).
Sartorious		Membrane filters. Battery time (6 h [100 L/m]−12 h [25 L/min]). Use of a variety of nutrient media. The solubility of the gelatin membrane filter allows further applications (e.g., rapid microbiology, virus sampling, and sampling of high bacterial concentrations). Sample volume—25, 50, 100, 250, 500, 750, and 1,000 L Air flow rate adjustable in five steps 10, 20, 30, 50, and 125 L/min (only when BACTairTM culture media plates are used).	Total sampling time with one battery charge approx. 4.5 h at 50 L/min, 4 h at 125 L/min.
Electrostatic precipitation		Flow rate = 1–10 L/min. Different collection media can be used in this study: agar, deionized and sterilized water, and a filter material.	Exposure of microorganisms to an external electrical field can cause conformational changes in the protein helix-coil or metastable conformational transitions in the polynucleotide mode. Some sensitive bacteria can sustain injuries during sampling. It was found that evaporation of the collection medium, such as water or the liquid component of agar, increases the humidity inside the EAS. This may affect the size distribution of the particles being collected and thus result in decreasing physical and biological efficiencies of the electrostatic precipitation method.

^*a*^ High efficiency particulate air (HEPA), referring to a type of mechanical air filter designed to capture at least 99.97% of airborne particles down to 0.3 µm in size, including dust, pollen, mold, bacteria, and other pollutants.

^*b*^ Electrostatic air sampler (EAS), a specific type of air sampler that uses electrostatic forces to attract and capture airborne particles, especially biological ones.

When selecting a bioaerosol sampling method, it is important to consider the specific environmental context and research goals. For urban settings, where particulate pollution is higher and microbial loads may be lower or more transient, high-volume samplers like the Coriolis Micro or SASS 3100 offer better temporal resolution and are well suited to short-duration, high-throughput sampling, including culture-independent analyses. However, their cost and sensitivity to field conditions may limit their use in rural or remote areas. Passive samplers and filtration-based methods, while low-cost and field-deployable, require long exposure times and may be more suitable for rural environments with stable conditions or where power sources are unavailable. Methods like electrostatic precipitation and impactors (e.g., Andersen samplers) provide fine particle separation and can be adapted for both culture-dependent and -independent analyzes, but often require careful calibration and humidity control. Importantly, compatibility with culture-independent methods such as DNA-based analysis varies: wet sampling (e.g., Coriolis Micro) generally preserves genetic material more effectively, while dry sampling or impact-based techniques may reduce microbial viability and degrade nucleic acids. These trade-offs should be carefully considered when designing studies across diverse environmental contexts.

Given the growing emphasis on culture-independent approaches in microbiome science, it is important to consider how sampling methods influence downstream molecular analyses. While our focus was on comparing air sampling strategies, many of the methods discussed vary in their compatibility with DNA/RNA-based techniques. Wet sampling approaches, such as impingement and the Coriolis Micro, are generally better at preserving nucleic acids, making them well-suited for amplicon or metagenomic sequencing. In contrast, dry sampling methods—such as impactors or filters—may reduce microbial viability and degrade genetic material, though they remain widely used due to field robustness and portability. These trade-offs should guide method selection when molecular analysis is a key objective.

### Reverse brainstorming

**Fig 3:**
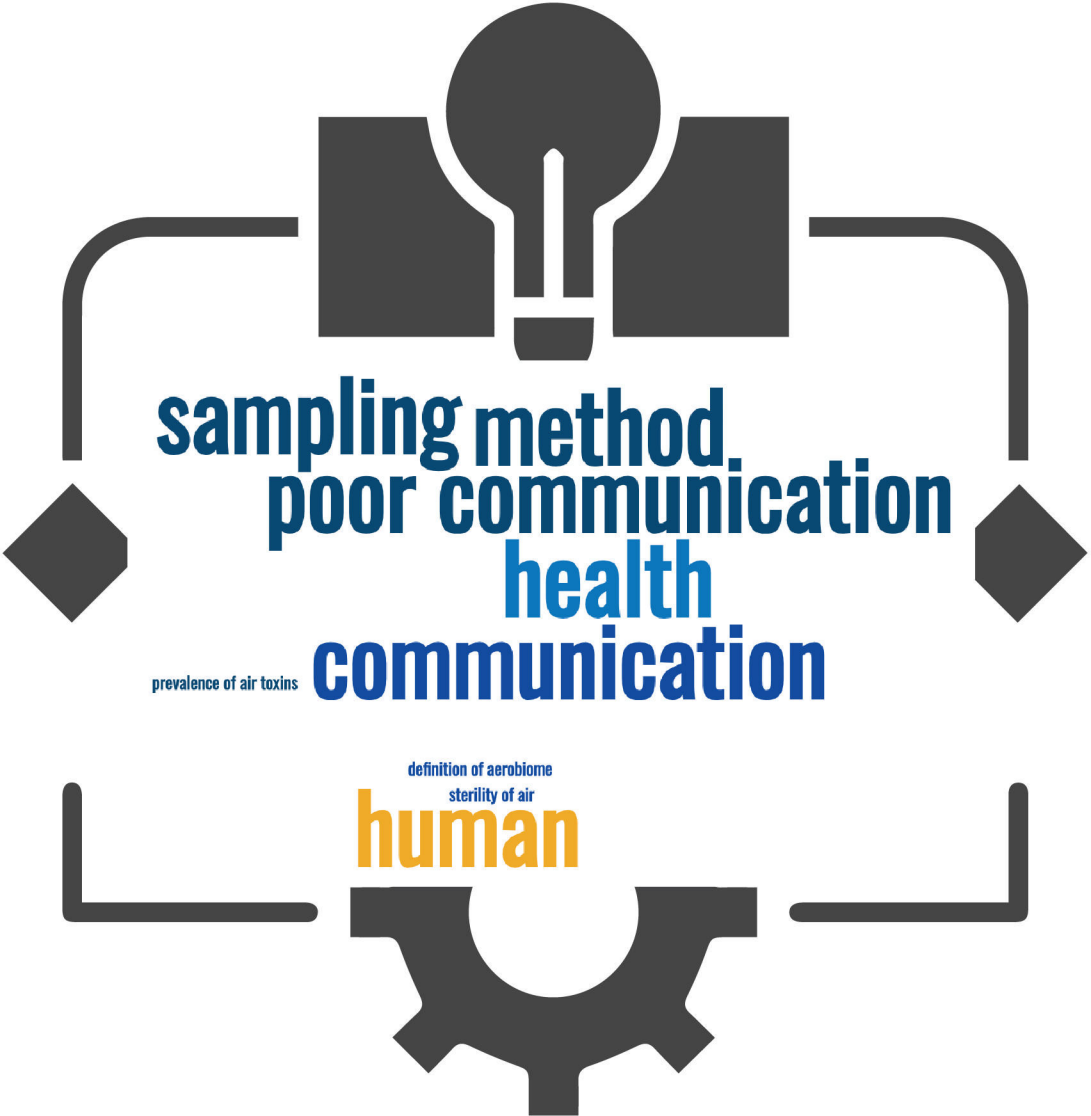
A word cloud of the key perceived issues to address to improve the development of aerobiome science. Themes include communication, methodology, and health concerns.

Reverse brainstorming is a creative problem-solving technique where instead of seeking solutions to a problem, participants focus on generating ideas to make the problem worse. By intentionally brainstorming ways to exacerbate the issue, individuals can identify potential pitfalls, obstacles, and negative factors that might not be immediately obvious. This approach can lead to a deeper understanding of the problem and highlight areas that need attention. Once these harmful ideas are listed, the process is reversed to find constructive solutions by addressing and mitigating the identified negative factors. Reverse brainstorming encourages lateral thinking and can uncover innovative solutions that might otherwise be overlooked (31). The workshop reverse brainstorming activity aimed to identify key factors (Fig. 3) that could inhibit the development of the AIR Hub’s objectives and co-design potential solutions (Table 3).

**TABLE 3:** Results of the reverse brainstorming activity

Problems	Exacerbated	Solutions
Definition of aerobiome and poor communication	Worsen the poor communication—generate confusing messages.	Definitions and recognition by the World Health Organization.
Sterility of air (fear and concern)	Poor communication—media misinformation; less research; disregard of beneficial components.	More research on the beneficial components and clearer messaging by scientists and policymakers.
Prevalence of air toxins/pollutants	Proliferation of pollution, ecosystem degradation.	Legislation responses on WHO regulations on air quality; sustainable development goals, including scientific literature on air quality.
Regulatory “nightmare”—separation of relevant stakeholders and sector fragmentation	Ignorant leader; lack of science-based policies.	Talk with policy leaders—via small but progressive steps.
Complexity—in aerobiome science, design, linking to human and ecosystem health	Conflicting evidence; lack of collaborations; too many variables.	Robust experimental designs.
Limitations in sampling and other methods	Do not use multiple methods; ignore limitations.	Use complementary methods; innovate new methods.
Methods—sampling dynamics	Extreme weather events; ignore variability.	Create standards; measure environmental conditions.
Funding	Not applying for grants; underestimate the benefits/risks of collaborations.	Apply for more grants and emphasize the need (funder awareness).
Vulnerable populations (health, socioeconomics)	Disconnect cause/effect; victim shaming; #dealwithit	Improve communication; create risk assessments.
One health (environment, humans, animals)	Implement localized solutions; disconnect; quick fixes.	Collaborate; learn from past efforts; acknowledge others (emotions, people, culture).
Methodological uncertainty (equipment, sampling, reproducibility)	Create silos; no standards; small sample sizes.	Outline problem framework—parameters; create standards.
Polarization and poor communication	Do not collaborate; lack of open data.	Involve the public, researchers, government, funders, and communicators in policy development.

## CONCLUSIONS AND POLICY IMPLICATIONS

Understanding the dual nature of bioaerosols—both harmful and beneficial—is essential for developing comprehensive strategies to improve public and ecosystem health. The recent paradigm shift in bioaerosol research emphasizes the need to harness the positive aspects of microbial communities in the air while continuing to mitigate their harmful effects. The Aerobiome Innovation and Research Hub (AIR Hub) represents an effort to advance this holistic perspective. This advancement will have implications for various international policies (Table 4).

**TABLE 4:** International policies relevant to advancing a holistic perspective in aerobiome science

Policies/guidelines	Relevance
World Health Organization (WHO) Air Quality Guidelines.	These guidelines provide recommendations on safe levels of various air pollutants, including particulate matter (PM_2.5_ and PM_10_), which are relevant to harmful bioaerosols. The guidelines aim to protect human health from the adverse effects of air pollution.
United Nations Sustainable Development Goals (SDGs)	SDG 3: Good health and well-being Targets under this goal include reducing deaths and illnesses from hazardous chemicals and air, water, and soil pollution and contamination. SDG 11: Sustainable cities and communities This goal includes making cities and human settlements inclusive, safe, resilient, and sustainable, with specific targets related to air quality. SDG 13: Climate action Aims to combat climate change and its impacts, which indirectly includes improving air quality and reducing harmful bioaerosols.
The Clean Air Act (U.S. Environmental Protection Agency)	This U.S. law regulates air emissions from stationary and mobile sources to ensure that air quality meets health-based standards. It includes regulations that limit emissions of harmful particles and gases, which are relevant to bioaerosol research.
European Union Air Quality Directive	The EU has set limits for air pollutants, including PM2.5 and PM10, through this directive. It aims to protect human health and the environment from the negative impacts of air pollution, which includes harmful bioaerosols.
Convention on Biological Diversity (CBD)	This international treaty aims to conserve biological diversity, promote sustainable use of its components, and ensure fair and equitable sharing of benefits arising from genetic resources. The convention’s goals indirectly support the understanding and management of bioaerosols as part of biodiversity.
The International Health Regulations (IHR)	Managed by the WHO, the IHR is a legally binding framework for international health security. It includes measures for preventing and responding to the international spread of diseases, including those transmitted by bioaerosols.
Kyoto Protocol and Paris Agreement	These international treaties aim to combat climate change by reducing greenhouse gas emissions. While primarily focused on climate change, they also address broader environmental health issues, including air quality improvement.
Nagoya Protocol on Access and Benefit-Sharing	A supplementary agreement to the CBD, this protocol addresses the fair and equitable sharing of benefits arising from the utilization of genetic resources, which can include microorganisms in bioaerosols.
World Health Organization (WHO) Guidelines on Indoor Air Quality	These guidelines provide recommendations for maintaining healthy indoor air quality, addressing pollutants that can affect health, including bioaerosols.
Global Action Plan for the Prevention and Control of Noncommunicable Diseases 2013–2020 (WHO)	This plan aims to reduce the burden of noncommunicable diseases through measures that include improving air quality and reducing exposure to harmful pollutants, including bioaerosols.

Current air quality frameworks, including the WHO Air Quality Guidelines, primarily target chemical pollutants and particulate matter, with limited attention to the biological composition of the air. However, there is a pressing need to evolve existing regulatory frameworks to incorporate the biological dimensions discussed in this article. This could involve expanding monitoring systems to include indicators of bioaerosol composition and function, particularly in relation to immune modulation, mental health, and biodiversity support. As highlighted by Kumar and Gupta (32), adaptive and science-informed air pollution policies are essential for responding to emerging threats and opportunities (32). Integrating beneficial bioaerosols into air quality regulation would align with a broader shift towards more holistic and health-promoting environmental policies (e.g., WHO-IUCN Nature-Based Solutions for Health) and support the development of evidence-based strategies that safeguard not only the absence of harm but also the presence of ecological benefit in the air.

By encouraging interdisciplinary collaborations and promoting innovative research, we can elucidate the potential roles of beneficial bioaerosols (airborne compounds and molecules that may confer a benefit, e.g., health-promotion) in human and ecosystem health. There is a need for diverse microbial exposures and effective communication and policy development to support these findings. Our tabular outputs evaluate air sampling methods, identify key factors that could inhibit the development of future aerobiome research, and set the initiative in the context of global policies. Moving forward, integrating knowledge of bioaerosols (both the salutogenic and pathogenic components) into environmental management practices and research agendas will be crucial for creating healthier, more resilient ecosystems and communities.

### Highlights

- Paradigm shift: aerobiomes play dual roles—harmful and beneficial bioaerosols can impact both ecosystem and human health.
- Holistic air quality: promoting beneficial bioaerosols while mitigating harmful ones can improve environmental health.
- Interdisciplinary collaboration: we need diverse teams to foster cross-sectoral innovation in aerobiome science and health.
- Policy integration: understanding bioaerosols supports global health and environmental policies, driving comprehensive change.

## References

[1] McCartney HA, Fitt BDL, Schmechel D. 1997. Sampling bioaerosols in plant pathology. J Aerosol Sci 28:349–364. doi:10.1016/S0021-8502(96)00438-7

[2] Li M, Wang L, Qi W, Liu Y, Lin J. 2021. Challenges and perspectives for biosensing of bioaerosol containing pathogenic microorganisms. Micromachines (Basel) 12:798. doi:10.3390/mi12070798 34357208 PMC8307108

[3] Nazaroff WW. 2016. Indoor bioaerosol dynamics. Indoor Air 26:61–78. doi:10.1111/ina.12174 25483392 PMC7165847

[4] Gonzalez-Martin C, Teigell-Perez N, Valladares B, Griffin DW. 2014. The global dispersion of pathogenic microorganisms by dust storms and its relevance to agriculture. Adv Agron 127:1–41. doi:10.1016/B978-0-12-800131-8.00001-7

[5] Younis F, Salem E, Salem E. 2020. Respiratory health disorders associated with occupational exposure to bioaerosols among workers in poultry breeding farms. Environ Sci Pollut Res Int 27:19869–19876. doi:10.1007/s11356-020-08485-x 32227302

[6] Gomes B, Dias M, Cervantes R, Pena P, Santos J, Vasconcelos Pinto M, Viegas C. 2023. One health approach to tackle microbial contamination on poultries—a systematic review. Toxics 11:374. doi:10.3390/toxics11040374 37112601 PMC10142658

[7] Walser SM, Gerstner DG, Brenner B, Bünger J, Eikmann T, Janssen B, Kolb S, Kolk A, Nowak D, Raulf M, Sagunski H, Sedlmaier N, Suchenwirth R, Wiesmüller G, Wollin K-M, Tesseraux I, Herr CEW. 2015. Evaluation of exposure–response relationships for health effects of microbial bioaerosols – a systematic review. Int J Hyg Environ Health 218:577–589. doi:10.1016/j.ijheh.2015.07.004 26272513

[8] Yoo K, Lee TK, Choi EJ, Yang J, Shukla SK, Hwang S-I, Park J. 2017. Molecular approaches for the detection and monitoring of microbial communities in bioaerosols: a review. J Environ Sci (China) 51:234–247. doi:10.1016/j.jes.2016.07.002 28115135

[9] Patterson B, Morrow C, Singh V, Moosa A, Gqada M, Woodward J, Mizrahi V, Bryden W, Call C, Patel S, Warner D, Wood R. 2017. Detection of Mycobacterium tuberculosis bacilli in bio-aerosols from untreated TB patients. Gates Open Res 1:11. doi:10.12688/gatesopenres.12758.2 29355225 PMC5757796

[10] Robinson JM, Breed MF. 2023. The aerobiome-health axis: a paradigm shift in bioaerosol thinking. Trends Microbiol 31:661–664. doi:10.1016/j.tim.2023.04.007 37211511

[11] Flies EJ, Jones P, Buettel JC, Brook BW. 2020. Compromised ecosystem services from urban aerial microbiomes: a review of impacts on human immune function. Front Ecol Evol 8:568902. doi:10.3389/fevo.2020.568902

[12] Robinson JM, Cando-Dumancela C, Antwis RE, Cameron R, Liddicoat C, Poudel R, Weinstein P, Breed MF. 2021. Exposure to airborne bacteria depends upon vertical stratification and vegetation complexity. Sci Rep 11:9516. doi:10.1038/s41598-021-89065-y 33947905 PMC8096821

[13] Li Q, Nakadai A, Matsushima H, Miyazaki Y, Krensky AM, Kawada T, Morimoto K. 2006. Phytoncides (wood essential oils) induce human natural killer cell activity. Immunopharmacol Immunotoxicol 28:319–333. doi:10.1080/08923970600809439 16873099

[14] Li Q. 2023. New concept of forest medicine. Forests 14:1024. doi:10.3390/f14051024

[15] Woo J, Yang H, Yoon M, Gadhe CG, Pae AN, Cho S, Lee CJ. 2019. 3-Carene, a phytoncide from pine tree has a sleep-enhancing effect by targeting the GABA_A_-benzodiazepine receptors. Exp Neurobiol 28:593–601. doi:10.5607/en.2019.28.5.593 31698551 PMC6844839

[16] Fayet-Moore F, Robinson SR. 2024. A breath of fresh air: perspectives on inhaled nutrients and bacteria to improve human health. Adv Nutr 15:100333. doi:10.1016/j.advnut.2024.100333 39486624 PMC11626012

[17] Langford B, Ryalls JMW, Mullinger NJ, Hayden P, Nemitz E, Pfrang C, Robins A, Touhami D, Bromfield LM, Girling RD. 2023. Mapping the effects of ozone pollution and mixing on floral odour plumes and their impact on plant-pollinator interactions. Environ Pollut 336:122336. doi:10.1016/j.envpol.2023.122336 37595729

[18] Jones JC, Fruciano C, Hildebrand F, Al Toufalilia H, Balfour NJ, Bork P, Engel P, Ratnieks FL, Hughes WO. 2018. Gut microbiota composition is associated with environmental landscape in honey bees. Ecol Evol 8:441–451. doi:10.1002/ece3.3597 29321884 PMC5756847

[19] Rook GAW, Lowry CA, Raison CL. 2013. Microbial ‘Old Friends’, immunoregulation and stress resilience. Evol Med Public Health 2013:46–64. doi:10.1093/emph/eot004 24481186 PMC3868387

[20] Roslund MI, Puhakka R, Grönroos M, Nurminen N, Oikarinen S, Gazali AM, Cinek O, Kramná L, Siter N, Vari HK, Soininen L, Parajuli A, Rajaniemi J, Kinnunen T, Laitinen OH, Hyöty H, Sinkkonen A, ADELE research group. 2020. Biodiversity intervention enhances immune regulation and health-associated commensal microbiota among daycare children. Sci Adv 6:eaba2578. doi:10.1126/sciadv.aba2578 33055153 PMC7556828

[21] Soininen L, Roslund MI, Nurminen N, Puhakka R, Laitinen OH, Hyöty H, Sinkkonen A, ADELE research group. 2022. Indoor green wall affects health-associated commensal skin microbiota and enhances immune regulation: a randomized trial among urban office workers. Sci Rep 12:6518. doi:10.1038/s41598-022-10432-4 35444249 PMC9021224

[22] Ma L-C, Zhao H-Q, Wu LB, Cheng Z, Liu C. 2023. Impact of the microbiome on human, animal, and environmental health from a One Health perspective. Sci One Health 2:100037. doi:10.1016/j.soh.2023.100037 39077043 PMC11262275

[23] Cieza A, Oberhauser C, Bickenbach J, Jones RN, Üstün TB, Kostanjsek N, Morris JN, Chatterji S. 2016. Health is not just the absence of disease …. Int J Epidemiol 45:586–587. doi:10.1093/ije/dyw063 27174840 PMC4864884

[24] De Sario M, Katsouyanni K, Michelozzi P. 2013. Climate change, extreme weather events, air pollution and respiratory health in Europe. Eur Respir J 42:826–843. doi:10.1183/09031936.00074712 23314896

[25] Kelly FJ, Fussell JC. 2020. Global nature of airborne particle toxicity and health effects: a focus on megacities, wildfires, dust storms and residential biomass burning. Toxicol Res (Cambridge) 9:331–345. doi:10.1093/toxres/tfaa044 32905302 PMC7467248

[26] Zhang D, Li H, Luo XS, Huang W, Pang Y, Yang J, Tang M, Mehmood T, Zhao Z. 2022. Toxicity assessment and heavy metal components of inhalable particulate matters (PM2.5 & PM10) during a dust storm invading the city. Process Saf Environ Prot 162:859–866. doi:10.1016/j.psep.2022.04.065

[27] Sigurnjak Bureš M, Cvetnić M, Miloloža M, Kučić Grgić D, Markić M, Kušić H, Bolanča T, Rogošić M, Ukić Š. 2021. Modeling the toxicity of pollutants mixtures for risk assessment: a review. Environ Chem Lett 19:1629–1655. doi:10.1007/s10311-020-01107-5

[28] Li D, Xie J, Wang L, Sun Y, Hu Y, Tian Y. 2023. Genetic susceptibility and lifestyle modify the association of long-term air pollution exposure on major depressive disorder: a prospective study in UK Biobank. BMC Med 21:67. doi:10.1186/s12916-023-02783-0 36810050 PMC9945634

[29] Makri A, Stilianakis NI. 2008. Vulnerability to air pollution health effects. Int J Hyg Environ Health 211:326–336. doi:10.1016/j.ijheh.2007.06.005 17719845

[30] BioAirNet. 2024. White paper on healthy air. Available from: https://bioairnet.co.uk/bioairnet-position-paper-on-bioaerosols-and-the-healthy-air-concept. Retrieved 11 Jun 2024.

[31] Evans N. 2012. Destroying collaboration and knowledge sharing in the workplace: a reverse brainstorming approach. Knowl Manage Res Prac 10:175–187. doi:10.1057/kmrp.2011.43

[32] Kumar R, Gupta P. 2016. Air pollution control policies and regulations, p 133–149. In Plant responses to air pollution

